# An Unresectable Eyelid Squamous Cell Carcinoma Treated With Concurrent Chemoradiation Therapy

**DOI:** 10.7759/cureus.80984

**Published:** 2025-03-22

**Authors:** Kenzo Ohara, Yuto Izumiya, Takahiro Inoue, Takumi Kumai, Miki Takahara

**Affiliations:** 1 Department of Otolaryngology, Head and Neck Surgery, Asahikawa Medical University, Asahikawa, JPN

**Keywords:** cisplatin, concurrent chemoradiation therapy, docetaxel, eyelid squamous cell carcinoma, unresectable skin cancer

## Abstract

Cases of advanced eyelid squamous cell carcinoma (ESCC) are rare because most patients with eyelid cancer visit hospitals when the cancer is in relatively early stages. However, utill date, no study has recommended the most suitable treatment method for unresectable advanced skin squamous cell carcinoma (SSCC). Herein, we report a case of unresectable ESCC that invaded the orbital contents and paranasal sinuses, and was treated with moderate-dose cisplatin and docetaxel chemotherapy with radiation therapy. Our treatment method may be a feasible option for patients with advanced SSCC.

## Introduction

Eyelid cancer accounts for 5%-10% of all skin cancers, and eyelid squamous cell carcinoma (ESCC) accounts for 5%-10% of eyelid malignancies that are generally painless and progress slowly. Complete surgical resection is the standard treatment for ESCC [1]. Treatments for advanced and unresectable skin squamous cell carcinoma (SSCC) remain controversial owing to fewer cases and insufficient evidence. Although only approximately 5% of SSCC cases develop nodal metastasis, patients with SSCC larger than 2 cm exhibit higher rates of nodal metastasis and disease-specific death [2,3]. In the treatment of unresectable head and neck squamous cell carcinoma, concurrent chemoradiotherapy (CCRT) with >200 mg/m^2^ of cisplatin is considered the gold standard [4-6]. However, the optimal dose of cisplatin for the treatment of unresectable SSCC remains unknown. Here, we report a case of unresectable ESCC that was successfully treated with CCRT.

## Case presentation

**Figure 1:**
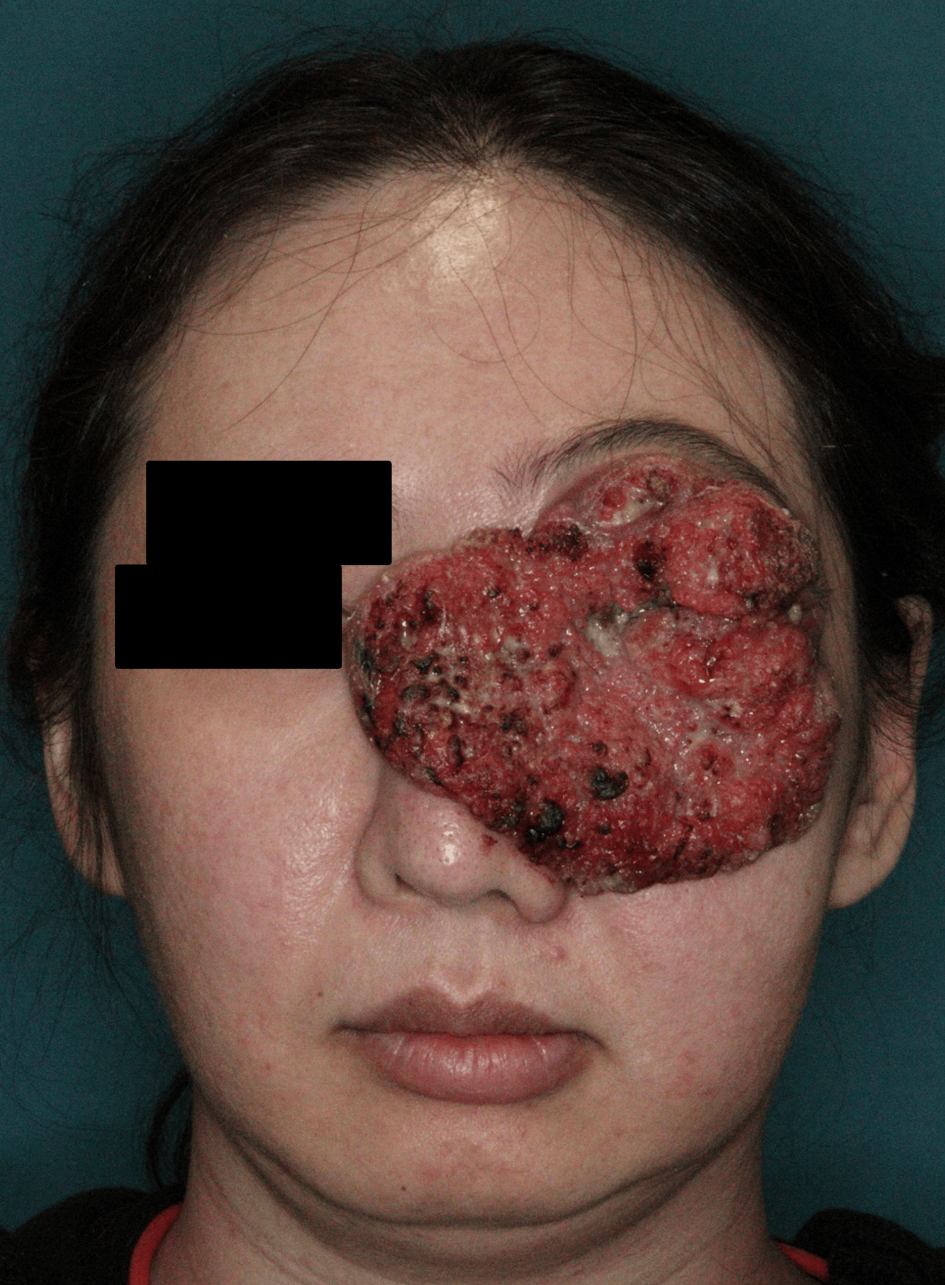
Tumor imaging before treatment The tumor covered most of the patient’s face

A 43-year-old woman presented with a tumor measuring >10 cm on the left side of her face. She had a history of depression and financial challenges that prevented her from seeking medical attention until the tumor had grown to cover one-third of her face (Figure 1).

**Figure 2:**
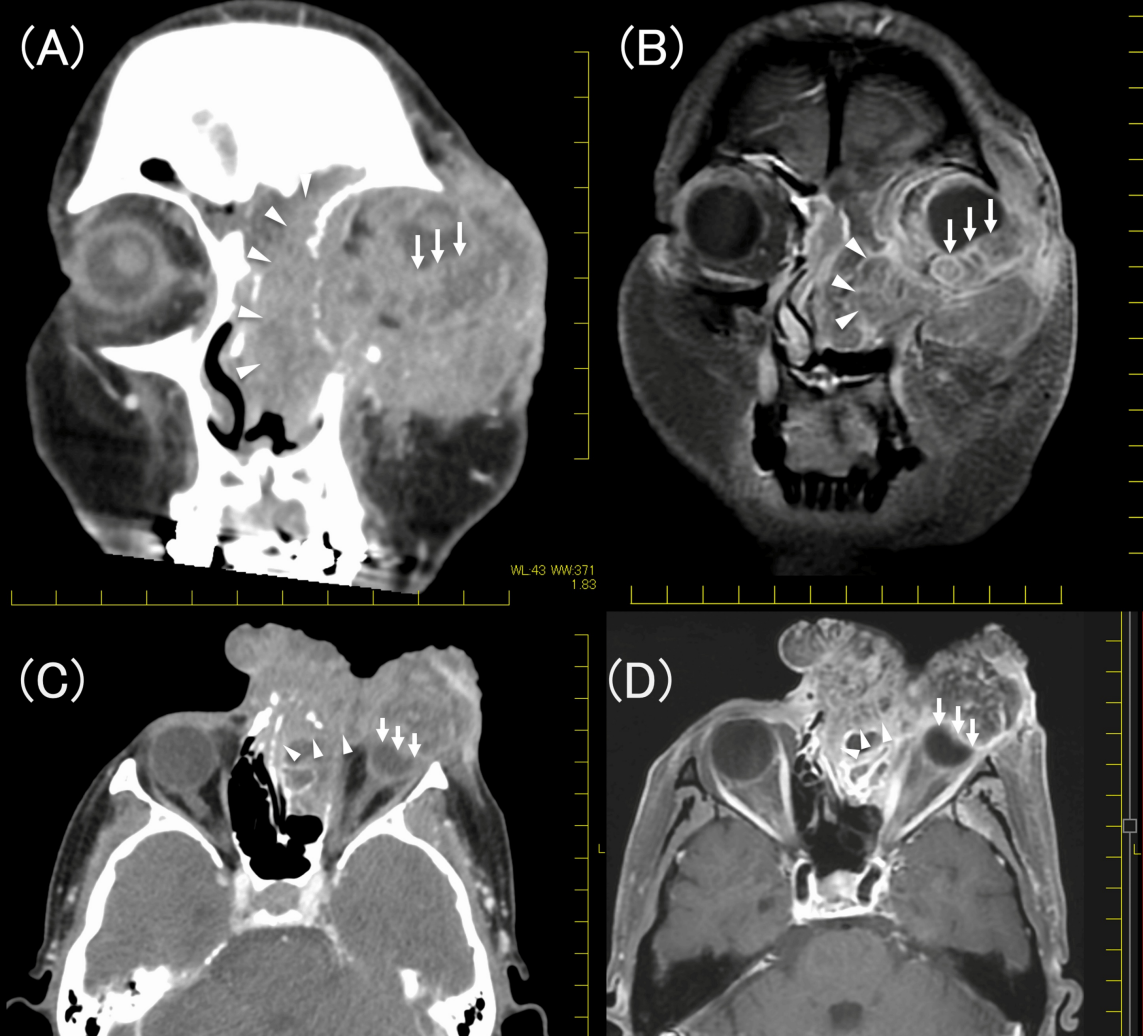
Computed tomography and magnetic resonance images. (A) Coronal contrast-enhanced computed tomography image. (B) Coronal T1-weighted contrast-enhanced magnetic resonance image. (C) Axial contrast-enhanced computed tomography image. (D) Axial T1-weighted contrast-enhanced magnetic resonance image The tumor invaded the orbital contents (white arrows) and sinonasal cavity (white arrowheads)

A biopsy confirmed that the tumor was a squamous cell carcinoma. Subsequent computed tomography (CT) and magnetic resonance imaging (MRI) revealed that the tumor originated from the left eyelid (Figures 2A-2D) and had metastasized to the left intraparotid lymph nodes and the left submandibular node with extracapsular extension.

**Figure 3:**
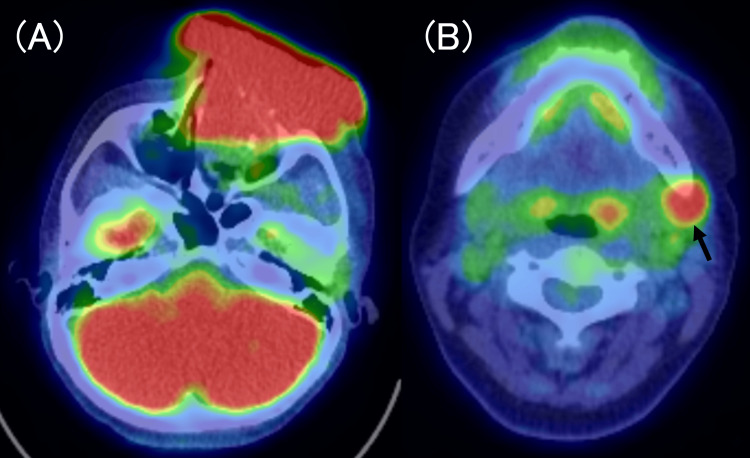
FDG-PET/CT scans. Elevated glycolytic activity is observed at the tumor site (A) and left submandibular lymph node (black arrow) (B) FDG-PET: fluorodeoxyglucose positron-emission tomography; CT: computed tomography

Further imaging revealed the invasion of the tumor into the orbital contents, frontal sinus, ethmoid sinus, and maxillary sinus. F-fluorodeoxyglucose positron emission tomography/CT (FDG-PET/CT) revealed no distant metastasis but indicated left submandibular metastasis (Figures 3A, 3B).

According to the eighth edition of the TNM Classification of Malignant Tumors by the Union for International Cancer Control, the patient was diagnosed with ESCC originating from the lower eyelid, T4bN2M0, Stage IIIB. The patient was referred to our hospital's dermatology department, but she was returned to the head and neck department. This is because, in Japan, compared with dermatology, head and neck medicine offers more treatment options (including cytotoxic anticancer drugs and immune checkpoint inhibitors) for squamous cell carcinoma that are eligible for medical insurance.

Although the tumor did not demonstrate intracranial extension, retro-orbital invasion, or invasion of major vessels, the patient presented with a metastatic left submandibular lymph node exhibiting extracapsular extension, suggesting that recurrence is likely to occur shortly after surgery. Furthermore, extensive resection with free flap reconstruction would significantly impair the patient’s quality of life. Therefore, head and neck surgeons, reconstructive surgeons, dermatologists, and radiologists deemed this case unresectable. The patient also declined surgical intervention, presumably owing to her underlying psychiatric condition and significant cosmetic disfigurement.

**Figure 4:**
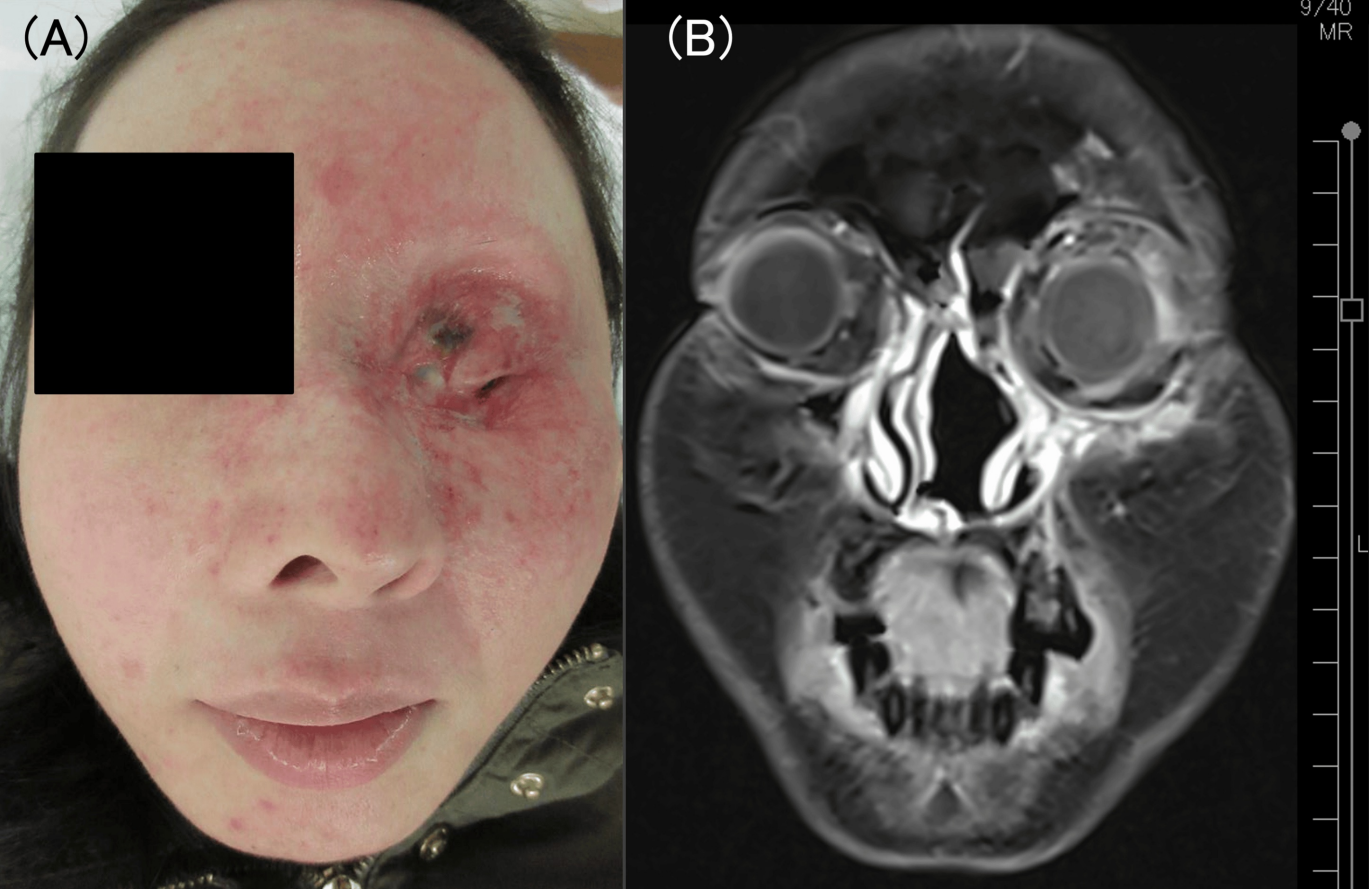
Patient’s face and MRI image 24 months after treatment. (A) The eyelid tumor completely disappeared; however, the patient lost eyesight in the left eye. (B) The coronal T1-weighted contrast-enhanced MRI showing complete remission MRI: magnetic resonance imaging

We administered CCRT comprising 180 mg/m^2^ cisplatin, 150 mg/m^2^ docetaxel, and 66 Gy intensity-modulated radiotherapy [7]. Specifically, docetaxel (50 mg/m^2^ on day 1) and cisplatin (15 mg/m^2 ^per day on days 2-5) were administered every three weeks for three cycles. Concurrently, the patient received 66 Gy at a dose of 2 Gy per fraction. Since the tumor was extremely large, we performed two replanning sessions not only to protect the other eye but also to maximize the efficacy of radiation therapy. The patient experienced no treatment-related toxicities of grade ≥3 during CCRT. FDG-PET/CT demonstrated complete remission three months after treatment, and regular imaging modalities (CT, MRI, or FDG-PET/CT) every three months revealed no tumor recurrence after 24 months. The patient facial appearance and MRI image 24 months after CCRT were shown in Figures 4A, 4B.

## Discussion

Advanced and unresectable ESCC is rare because the tumor is detected at early stages in most patients [8,9]. Therefore, no evidence-based treatment methods have been reported for unresectable ESCC. CCRT is considered the gold standard for the treatment of unresectable head and neck squamous cell carcinoma due to its superior efficacy compared to radiotherapy alone [10,11]. Herein, we describe a rare case of unresectable ESCC treated by CCRT.

In this case, definitive radiotherapy was applied exclusively to the primary tumor and not to cervical lymph node metastases, as primary tumor control was considered paramount. If residual cervical lymph node metastases persisted after CCRT, neck dissection was planned. Given the extremely large tumor size, two replanning sessions were performed to preserve right-eye vision and maximize the efficacy of radiation therapy.

At our institution, we generally use both a triweekly regimen of 100 mg/m² cisplatin and a triweekly regimen of 60 mg/m² cisplatin combined with 50 mg/m² docetaxel [7]. Although we have not conducted a prospective study to confirm the efficacy of the triweekly 60 mg/m² cisplatin and docetaxel regimen over the triweekly 100 mg/m² cisplatin regimen, the former is often selected for patients with a higher likelihood of residual tumor after primary treatment to help preserve renal function. For patients with tumor recurrence, we have two options for cisplatin administration: a triweekly regimen of 100 mg/m² [12] or a weekly regimen of 40 mg/m² cisplatin [13].

Regarding the use of docetaxel as a radiosensitizer in patients with head and neck squamous cell carcinoma, a phase III study demonstrated its effectiveness even when radiotherapy was combined with docetaxel alone [14]. The maximum docetaxel dose in the study was 105 mg/m², suggesting that our CCRT regimen may be sufficient to control unresectable ESCC. Additionally, we successfully preserved renal function, allowing for the possibility of future retreatment with cisplatin. Although Japan has a good universal health system that allows patients to access medical care easily, the patient had both mental illness and financial concerns that prevented her from seeking medical care earlier. Even though our chemotherapy regimen did not contain >200 mg/m^2^ of cisplatin [7], we opted for it because of the extremely advanced tumor and lymph node metastasis with extracapsular extension. Since cisplatin causes cumulative nephrotoxicity [15], we attempted to preserve renal function to allow for cisplatin-based retreatment in the event of tumor relapse. Contrary to our expectations, the patient achieved complete remission after 24 months of treatment. If we detect tumor recurrence in the future, we plan to administer a cisplatin-based regimen because we expect that the tumor will be cisplatin-sensitive and the patient will exhibit good renal function.

## Conclusions

Patients with unresectable ESCC require multidisciplinary therapy. Despite receiving a cisplatin dose of <200 mg/m^2^, the patient has remained in complete remission for 24 months following treatment. Our CCRT regimen with moderate-dose cisplatin and docetaxel may be a good option for the treatment of unresectable head and neck SSCC.
